# Polarized light based scheme to monitor column performance in a continuous foam fractionation column

**DOI:** 10.1186/1754-1611-4-5

**Published:** 2010-04-15

**Authors:** Janakiraman N Swamy, Czarena L Crofcheck, MP Mengüç

**Affiliations:** 1 Biosystems & Agricultural Engineering, 128 CE Barnhart Building, University of Kentucky, Lexington, KY 40546, USA; 2 Mechanical Engineering, University of Kentucky, 269 Ralph G. Anderson Building, Lexington, Kentucky 40506, USA

## Abstract

**Background:**

A polarized light scattering technique was used to monitor the performance of a continuously operated foam fractionation process. The *S*_11 _and *S*_12 _parameters, elements of the light scattering matrix, combined together (*S*_11_*+S*_12_) have been correlated with the bubble size and liquid content for the case of a freely draining foam. The performance of a foam fractionation column is known to have a strong dependence on the bubble size distribution and liquid hold up in foam. In this study the enrichment is used as a metric, representative of foam properties and column performance, and correlated to the *S*_11_*+S*_12 _parameter.

**Results:**

Three different superficial gas velocities (6.9, 7.5, and 10.6 cm/min) and four different pH values (4.8, 5.5, 6.5, and 7.5) are tested for the foam fractionation of a dilute solution of bovine serum albumin (0.1 mg/ml). As a result, at scattering angle of 125° the magnitude of *S*_11_*+S*_12 _is higher as the pH increases. When the bubble sizes are small with a larger liquid content, the foam is strongly back scattering resulting in lower values of *S*_11_*+S*_12 _(at 125°) at pH = 4.8. The light scattering data and the enrichment values are measured over a period of 90 minutes and correlated using a linear model. The predictive power of the model was found to be statistically significant.

**Conclusion:**

The time average *S*_11_*+S*_12 _shows a direct proportionality with the enrichment value, indicating that polarized light should be a valuable technique for monitoring foam fractionation columns. Additional knowledge of the nature of dependence between foam properties and *S*_11_*+S*_12 _combined with models relating the enrichment to the bubble size and liquid hold up is needed to develop an accurate diagnostics tool for monitoring enrichment utilizing *S*_11_*+S*_12 _measurements.

## Introduction

Foam fractionation is a separation technique in which the surface active solutes are concentrated from very dilute solutions by preferential adsorption at the gas-liquid interface as a gas is sparged through the solution. The process is very similar to froth flotation which has been a very common practice in the mining industry for the concentration of mineral ores [1] and is sometimes referred to as foam flotation [2]. Schütz [3] was the first to use foam fractionation to concentrate cholic acid from its mixture with sodium cholate. Later in 1959, Schnepf and Gaden [4] used this process to concentrate bovine serum albumin (BSA) from a dilute aqueous solution. Since then a large number of investigations have been published on foam-based protein/enzyme separation and/or purification [5-18].

Protein adsorption correlates with surface activity and the unfolding of the protein at the gas-liquid interface which is governed by surface hydrophobicity and surface tension [15]. The most commonly used techniques for separation and/or purification of proteins are ion exchange, ultra-filtration, and precipitation. Foam fractionation offers attractive advantages over these techniques in terms of capital and operation costs [8,11,13]. Further, the process is simple in operation and easy to scale-up [11]. The potential application of this method lies in the early stages of a downstream purification, for separation and concentration of proteins from a large volume of crude starting material.

Various investigations have shown that batch and continuous foam fractionation can be used to concentrate proteins from their dilute solutions [5,6,9,13,16,17]. Brown et al. [6] highlighted the effects of feed concentration, feed/gas flow rates, bubble size, pH, and ionic strength on the enrichment and recovery of continuously foamed BSA. Uraizee and Narsimhan [7] extended this work by including the effect of pool height and coalescence on column performance. Brown et al. [18], for the first time, presented a statistical analysis of the process variables and first order interactions on the performance parameters in continuous foaming of β-casein. The interactive effect of the process variables is particularly important in the determination of optimum process conditions. Recently, Linke et al. [13] varied the superficial gas velocity (SGV), pH value, and the surfactant choice to optimize the separation of esterase secreted by *P. sapidus*.

Liquid drainage refers to the flow of liquid between the bubbles in a foam layer. Significant liquid drainage can improve the enrichment of foamate, but it can also lead to foam instability and bubble coalescence [18]. Column parameters such as SGV, bubble size (column frit size), foam height, and liquid pool height influence protein foamability and foam stability. A high SGV increases the number of bubbles, hence increasing the surface area available for protein adsorption, which can enhance both enrichment and recovery; but at the same time, the associated increase in liquid holdup reduces enrichment and increases recovery. Large velocities can also lead to bubble coalescence and protein denaturation from the buildup of shear forces [7,9]. A low SGV reduces protein recovery and increases enrichment by lowering the rate of liquid uptake and increasing liquid drainage, producing a drier but more concentrated foam [5,7,18]. Likewise, large bubbles improve liquid drainage, generating a higher enrichment and reduced recovery. Small bubbles provide a greater overall interfacial area available for adsorption and liquid holdup. Liquid holdup depends on the properties of the solution, the bubbles size and the SGV [19]. Each of these effects on the foam dynamics is reflected through changes in the bubble size distribution and liquid content. In essence, all of the chemical and physical factors express their influence in some form through the bubble size distribution and liquid content. Thus, measurement of these parameters can provide for a direct means to control the process conditions for an optimum performance.

Only a few studies on the measurement of bubble size and liquid hold up in relation to foam fractionation exist in the literature. Uraizee and Narsimhan [7] measured the changes in bubble size distribution as a function of foam height for a continuous foaming column using video imaging and reported that larger bubbles were produced at lower gas flow rates and lead to higher enrichments. This was attributed to enhanced coalescence effects causing an accelerated drainage. Recovery was found to be higher for foam composed of smaller bubbles produced at higher gas flow rates. Lockwood et al. [20] studied the fractional liquid hold up in the foam layer as a function of column height using gamma-scintigraphy. It was seen that porous frits with smaller pore sizes produce slow draining foams. Stevenson et al. [21] used nuclear magnetic resonance imaging (NMRI) to measure liquid holdup and drainage. Wong et al. [22] measured the bubble size in a continuous system using digital photography to calculate the interfacial area in foam and related it to the enrichment and recovery. It was reported that smaller bubbles produced at lower gas flow rates close to the isoelectric point of the protein provided the highest enrichment. Du et al. [23] measured the bubble size distribution in a continuous foam fractionation column using a photoelectric probe technique. It was observed that log-normal distributions best approximated the bubble size distribution across the cross section of a foam fractionation column. In a later study, using the same scheme they investigated the influence of bubble size on column performance [23]. The results reported were in agreement with findings of Uraizee and Narsimhan [7]. Varley et al. [24] investigated the use of a conductivity probe for predicting changes in liquid holdup. However, Rosa et al. [12] decided to use a photographic method for measuring the bubble size, for simplicity.

While the measurement techniques used for the studies mentioned above provide for relationships between process conditions, bubble size/liquid hold up, and performance, they are not suitable for developing diagnostic schemes for continuous monitoring and control of column performance. Further, the direct imaging methods (i.e. photography and video imaging) only provide for measurement of bubbles on the surface, while, photoelectric probes are intrusive to the process. Hence, there is a well motivated need for development of fast, continuous, and non-intrusive methods for monitoring the bubble size distribution and liquid hold up in the foam layer. Optical techniques have immense potential for obtaining such information in a non-invasive way.

Multiple light scattering measurements have been used to probe the structure of foams [25]. Using such techniques Durian and collaborators studied the transient dynamics of foams treating light as a diffusing wave. The diffusion coefficient was used to describe the average bubble size and liquid fraction as a function of time. The depolarization effects due to bubble size and polydispersity in foams has also been investigated [26-28]. It has been observed that the attenuation in intensity combined with the polarization data is sensitive to bubble size and liquid fraction.

## Polarized Light Scattering in Foams

In polarized light scattering, the variation in polarization of the incident light due to scattering is measured in addition to the attenuation in intensity. This technique has been useful in characterization of dense media [29,30]. Stokes' vector can be used to fully describe the intensity and polarization state of light and can be expressed using the parallel and perpendicular components of the electric field. The Stokes' vector corresponding to incident and scattered light is related through the Mueller matrix (also known as the scattering matrix, 4 × 4) which provide for a complete description of the optical properties of the media, given as [31]:(1)

Out of the sixteen scattering matrix elements (*S*_*ij*_'s), only six of these elements are independent (*S*_11_, *S*_12_, *S*_22_, *S*_33_, *S*_34_, and *S*_44_). Measurement of these six elements is usually enough to describe the scattering properties of the media. For the case of foams, the specific elements *S*_11_, *S*_12_, and *S*_33 _at certain back scattering angles (120°-135°) have been shown to have a good correlation with the foam properties [28]. To obtain all of these parameters at a given angle, a total of four measurements are required making continuous diagnostics difficult. But the four individual measurements which are combinations of these elements (such as I_H _= *S*_11_*+S*_12_, I_V _= *S*_11_-*S*_12_, I_P _= *S*_11_*+S*_33_, and I_M _= *S*_11_-*S*_33_) have been found to be sensitive to variation in bubble size and liquid hold up for dynamic foams [27]. Specifically the *S*_11_*+S*_12 _parameter is found to increase with an increase in bubble size and decrease in liquid fraction, and vice-versa. Hence, the parameter (*S*_11_*+S*_12_) is considered for this study.

## Objective

The objective of this study is to investigate the utility of polarized light scattering to monitor the column performance in a foam fractionation column via measurement of foam properties. Analytical expressions describing the multiple scattering of light in a highly scattering media such as foam are difficult to obtain [32]. Hence, direct correlation of measured intensity to the size distribution data is usually not possible without elaborate calibration schemes requiring several carefully designed control experiments and accurate numerical predictions. Relationships between the foam properties and performance are difficult to quantify due to the difficulty in determining the bubble size distribution. Hence, in the present study, enrichment is treated as a parameter representative of the foam properties as it is directly dependent on the bubble size and liquid hold up. If the light scattering data can be correlated to enrichment, then a diagnostic scheme can be developed for continuous monitoring of the process. It is important to note here that although enrichment is being used as a representative variable, the light scattering measurements are consequences of the bubble size distribution and liquid content of the foam layer.

A semi-batch/continuous mode of operation is considered for the study. Foam fractionation is performed on dilute solutions of bovine serum albumin (BSA) as it has been used by several researchers as a model protein to study foam fractionation [2,7]. BSA, a globular protein, produces stable foams in comparison to flexible proteins such as β-casein [2]. Preliminary experiments are conducted at a range of pH and SGV values to investigate the performance of the foam fractionation column as a function of time. Based on these experiments, specific pH values, SGV, and time period are selected for the light scattering experiments. The enrichment and the parameter *S*_11_*+S*_12 _are measured as a function of time for each pH and SGV used. Correlation between the enrichment and light scattering data is obtained and its predictive power is analyzed. Finally, the process is perturbed by varying the SGV in steps and the response in the light scattering signal is analyzed in relation to foam properties and enrichment.

## Materials and methods

### Experimental Setup

**Figure 1:**
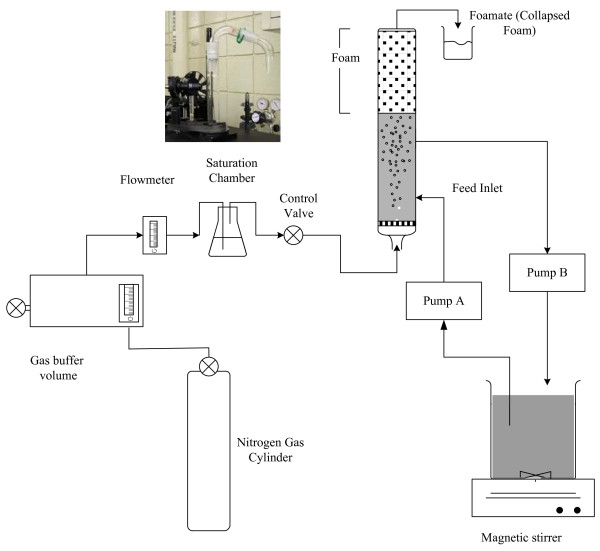
**Schematic of the continuous foam fractionation set up used for experiments**.

Foam fractionation experiments were performed using the set up shown in Figure 1. The foaming column is approximately 50 cm tall with an internal diameter of 2.54 cm and an external diameter of 2.7 cm. The column was made in the University of Kentucky glass shop where the top and bottom of the column were fabricated to be compatible with standard glassware from Ace Glass. The top of the column allows for the insertion of a glass elbow (Ace Glass, 5070/9052 Adapter, 75 degree angle with 24/40-24/40 joints (Part # 5070-10)) to direct the foam into the collection cup. The bottom of the column can be closed using a threaded hollow joint (Ace Glass, 9083/5080 Adapter, Distillate Take-Off with 24/40 joint, 105 degree angle (Part # 5080-10)) which allows for mounting a porous sintered glass frit (Ace Glass) on one end and gas inlet on the other. Two ports serve as inlet and outlet for recirculation of the protein solution from a 2000 ml flask which serves as a reservoir and is continuously stirred using a magnetic stirrer. A peristaltic pump (pump A) serves to feed the column from the reservoir, while pump B (Fisher Scientific) drains the column solution into the reservoir. Nitrogen gas saturated with moisture is sparged through the porous frit for generation of bubbles, and the foam is collected through an overflow tube (bent downwards at an angle of 45°) mounted on the top of the column. The gas is passed through a buffer volume, to minimize variations in the gas flow rate due to pressure fluctuations in the gas cylinder. The flow rate is adjusted to a desired value using the flow valve on the cylinder and the rotameter knob. The flow rate is accurately measured within 1% using an Omega digital flow meter.

**Figure 2:**
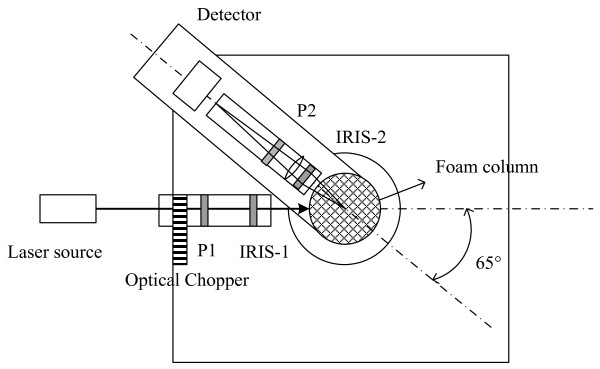
**Schematic of the light scattering set up used to monitor the column performance over time**.

Light scattering experiments were carried out with the setup shown in Figure 2. Optical components include two polarizers (P1 and P2), used to modulate incident and scattered light so that the polarized light scattered by foam layer in the column could be measured. An 8 mW helium neon laser (λ = 670 nm) is employed as the light source which was chopped at a frequency of 130 Hz using an optical chopper. The polarizer (P1) is fixed at 45° to set the Stokes' vector of the incident light. Scattered light that passed through P2 is detected using a photomultiplier tube (PMT; Hamamatsu H928) using a lock-in amplifier. Based on the detected intensity, the PMT provides a current signal which is converted to a voltage output using a trans-impedance pre-amplifier (Hamamatsu, C6648) which is measured at the chopping frequency (i.e. 130 Hz) using a dual phase lock-in amplifier (Scitec Instruments, UK). The purpose of using an optical chopper and lock-in amplifier is to eliminate noise from other sources, by modulating the input at a given frequency and then measuring the output at the same frequency with or without a phase difference.

### Experimental Procedure

Protein solutions were made using bovine serum albumin (BSA) procured from Sigma-Aldrich and used without further purification. A measured amount of protein powder was added to a known volume of distilled water with an appropriate buffer to produce solution of desired concentration. The pH of the solution was maintained using either Bis-Tris buffer (pH range 4.5-6.5) or Tris buffer (pH range 7-8). The pH was measured using a pH meter (Orion) and adjusted to the desired value by addition of acid (hydrochloric acid 0.01 M) or base (sodium hydroxide 0.01 M). A small amount of the protein solution was preserved to measure the initial protein concentration. The protein concentration was measured using the Bradford assay (Sigma Aldrich), with absorbance measurements taken on a Varian 300 UV-VIS spectrophotometer. The assay protocol provided by the manufacturer was followed for protein concentrations in the range 0.1-1.0 mg/ml. The procedure was slightly modified to allow for concentration measurements below 0.1 mg/ml.

The protein solution of desired concentration was poured into the reservoir. Pump A was used to transfer the solution into the column. Once the solution reached a desired height, pump B was also started (operating at the same flow rate as pump A) to allow for recirculation of the solution. Nitrogen gas was introduced into the column through the porous frit, at the desired gas flow rate. As foaming began, the inlet flow rate (pump A) was increased slightly to compensate for the liquid carried away by the foam. This slight increase in the feed rate was measured and optimized through independent experiments for a given combination of pH, SGV, and desired pool height. The pool height was maintained within ± 0.5 cm for all experiments. The overflowing foam was collected in a beaker at fixed periods of time from the inception of overflow. The foam was allowed to collapse and the resultant volume was measured using a measuring cylinder. The Bradford assay was used to quantify the protein concentration and the enrichment ratio (E) and recovery (R) are computed using the following relationships:(2)

Where, *C*_*f *_and *V*_*f *_are the protein concentration (mg/mL) and volume (mL) of the foamate. *C*_*i *_and *V*_*i *_are the initial protein concentration and volume of the liquid pool. Enrichment is defined as the ratio of the concentration of protein in foamate to that of the initial solution.

The light scattering set up was verified for alignment by filling the column with water and looking at the retro-reflection of the laser beam. The water was then completely drained and the solution was introduced for foaming as described earlier. When the foaming process began, all the instrumentation related to the light scattering measurements was turned on. The polarizer (P1) on the source side was fixed at 45° and the P2 was fixed at 0° (i.e. horizontal polarizer) orientation, with respect to the plane of detection. The detection optics mounted on the rotating stage were fixed at an angle of 125° relative to the direction of incident beam. When the foam crossed over the detection plane, the time average gate of the lock-in amplifier was adjusted to provide the highest intensity values. As the foam begins to overflow, the intensity data was acquired through the data acquisition system and recorded in the PC throughout the course of the experiment.

### Experimental Approach

The enrichment and recovery are inherently dependent on the bubble size distribution and liquid hold up in the foam layer [7]. It has been observed that as the bubble size increases in relatively drier foam, the enrichment ratio increases, while the recovery decreases. For foam with higher liquid content, the enrichment is usually low with higher recovery due to the larger volume of the foamate. Thus, the process parameters such as pH, gas flow rate, pool height, and initial concentration influence the foam properties, thereby influencing the performance. To understand the correlation between light scattering measurements and the foam properties, enrichment and recovery can serve as representative metrics. For that to be the case, it is important to study the effect of process variables on the performance for given foam fractionation set up.

Prior to the light scattering study, a set of experiments were conducted to study the effect of pH (4.5, 5.5, 6.5, and 7.5) and SGV (6.9, 7.5, and 10.6 cm/min) on enrichment and recovery for a constant feed solution concentration of 0.1 mg/ml and pool height of 35.5 ± 0.5 cm. Each experiment was conducted for a period of 150 minutes and the foamate was collected every 30 minutes from the beginning of overflow. The protein concentration was estimated and the performance indices computed. The primary objective of these experiments was to identify the time range for which the foam properties are near constant on an average, which in turn should result in relatively constant enrichment and recovery values per time interval. Each treatment combination was tested in triplicate.

From the foam fractionation experiments, the time period for light scattering experiments was selected based on relatively constant enrichment and recovery values for the pH and gas flow rate values considered. To investigate the effects of pH and flowrate on foam properties, light scattering experiments were performed for three solution pH values at a constant gas flow rate and for three gas flow rates for a constant solution pH. The specific flow rate and pH values were selected based on the enrichment and recovery observed in the foam fractionation experiments. Throughout each experiment the foamate was collected at 15 minute intervals and the corresponding volume and concentration was measured. The incident and detection plane was set at 3 cm from the top of the column, just before the foam overflow. This is done to minimize the differences in the properties of the foam measured via light scattering and the actual foam collected (used for volume and concentration measurements).

To further investigate the sensitivity of light scattering measurements on the foam properties, the processing conditions were perturbed. The process was operated at a set pH value of 5.5 and the flow rate was changed at 15 minute intervals. The intensity readings were taken for the entire 90 minutes of the experiment. The objective was to force a variation in foam properties in steps and observe the resulting response in the light scattering signal.

## Results and Discussion

### Effect of pH and Flow Rate on Performance

**Figure 3:**
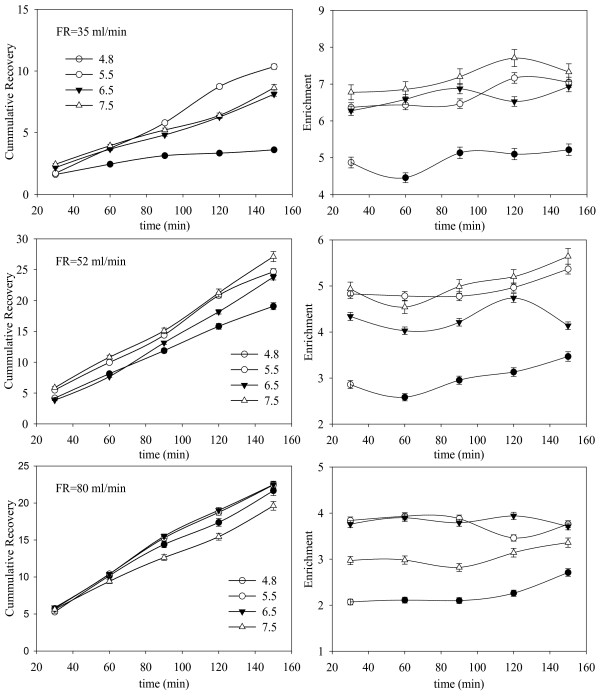
**Effect of pH and superficial gas velocity on the cumulative recovery and enrichment**. Data shown for a period of 150 minutes. The error bars are based on standard error (n = 3).

The effect of pH and superficial gas velocity on enrichment and recovery can be seen in Figure 3. The enrichment is found to be higher for pH values farther from the isoelectric point of BSA (pH~4.8). At the isoelectric point, protein is least soluble in water leading to high foamability (i.e. foam is easy to form, does not necessarily mean the foam is stable). As a result the bubbles are smaller with a high liquid content leading to lower enrichment values [7]. At all flow rates considered, the pH dependence of enrichment is not strictly monotonic. At the SGV of 6.9 cm/min, the enrichment values increase with pH initially, while a crossover in enrichment is observed for solution pH of 5.5 and 6.5. For a SGV of 7.5 cm/min, higher enrichments are observed at pH of 5.5 and 7.5 in comparison with pH of 6.5 and 4.8 (isoelectric point), while at 10.6 cm/min higher enrichment values are observed at pH of 5.5 and 6.5. The enrichment values are consistent for the initial period of 90 minutes, thereafter some variability is observed. This can be attributed to the fact that the concentration of the liquid pool begins to decrease as the protein is being recovered. The variation in the enrichment for the time period 90 to 150 minutes is different for different pH values. It is important to note that the influence of pH on enrichment is difficult to model and the correlations are usually qualitative in nature [2,6]. Enrichment shows a strong dependence on the gas flow rate for all pH values considered. As the gas flow rate increases the enrichment decreases due to larger volumes of liquid carried by the foam at a higher gas flow rate. Smaller size bubbles are observed as the gas flow rate increases, slowing down the drainage process and contributing to lower enrichment values. Further, at lower gas flow rates the bubble residence time is higher leading to enhanced adsorption [5-7].

The sensitivity of recovery to pH is higher at 6.9 cm/min and begins to decrease as the flow rate increases. Recovery is dependent on the volume of the foamate as well as enrichment. At higher flow rates, although the enrichment is lower, the foam overflow rate and the liquid hold up are higher [19] leading to higher recovery with little variation over different pH values [6,7]. On the other hand, for lower gas flow rates, enhanced adsorption kinetics and larger bubble sizes lead to higher enrichments, but the volume of foam recovered is small leading to lower recovery values. A constant rate of increase in the cumulative recovery is observed for most of the time at all pH values for SGV of 7.5 cm/min and 10.6 cm/min, while at 6.9 cm/min this is true only for the initial 90 minutes. An important inference to be drawn from this observation is that, when residence time of the bubbles is long (i.e. long enough not to be the limiting factor for adsorption), pH influences the protein removal and adsorption. This effect can be further investigated by considering a large range of flow rates and liquid pool heights for a constant foam height, but is beyond the scope of this study.

With light scattering experiments in mind, the important conclusions to be drawn from these experiments are: a) under the operating conditions considered, the column operates in steady state for the initial 90 minutes resulting in consistent recovery and enrichment; b) at pH = 5.5 enrichment is observed to be most consistent over the 90 minute interval for all the three flow rates considered; and c) SGV of 7.5 cm/min provides for a monotonically increasing variation in enrichment with pH for the initial period of 90 minutes. Additionally, the effects of gas flow rate and pH on the foam properties and/or enrichment/recovery are different in nature. Flow rate seems to have a more direct influence on the foam layer and hence the performance, while pH dependence seems to be indirect in terms of adsorption and interfacial properties of the bubbles.

### Variation in Light Scattering Data

Based on the above observations, a period of 90 minutes was selected as the time period for collecting light scattering data. Three pH values (4.8, 5.5, and 7.5) were considered at a fixed SGV of 7.5 cm/min and three SGV (6.9, 7.5, and 10.6 cm/min) were considered at a fixed pH of 5.5. This was done in order to separate the effects of gas flow rate and pH on enrichment which, as mentioned earlier, are different in nature.

**Figure 4:**
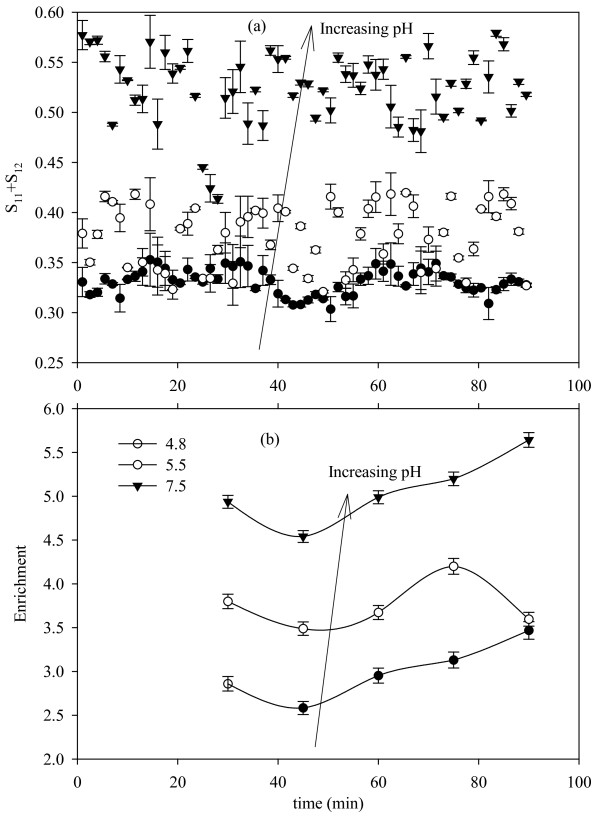
**Observed *S*_11_*+S*_12 _data and enrichment ratios as a result of pH variation**. The superficial gas velocity was 7.5 cm/min and the enrichment ratios were measured at 15 minute intervals. Error bars are based on the standard error (n = 3).

Figure 4a shows the variation of *S*_11_*+S*_12 _as a function of time for the three pH values considered (pH = 4.8, 5.5, and 7.5). It is observed that *S*_11_*+S*_12 _increases with increasing pH. The enrichment values measured over 15 minute intervals (starting at *t *= 30 minutes) show a similar dependence on pH. Since the scattering data corresponds to the foam properties, the inferences drawn from the data are also based on foam properties. From the observations discussed above and the findings of Uraizee and Narsimhan [7], the increase in enrichment with pH can be attributed to the larger bubble size (due to coalescence) resulting in faster drainage characteristics at pH values farther from the isoelectric point (pH~4.8). For a constant column diameter, the concentration of scattering sites decreases with increasing bubble size and decreasing liquid content. As a result at scattering angle of 125° the magnitude of *S*_11_*+S*_12 _is higher as the pH increases. This is in agreement with the scattering behavior observed in Swamy et al. [28] for the case of shaving foam. When the bubble sizes are small with a larger liquid content, the foam is strongly back scattering resulting in lower values of *S*_11_*+S*_12 _(at 125°) at pH = 4.8.

**Figure 5:**
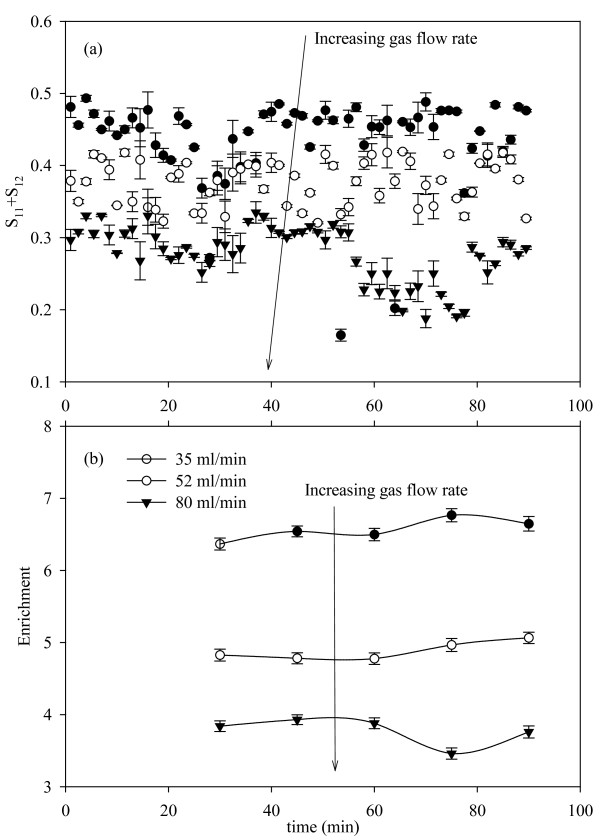
**Observed *S*_11_*+S*_12 _data and enrichment ratios as a result of variation in superficial gas velocity**. The pH was 5.5 and the enrichment ratios were measured at 15 minute intervals. Error bars are based on the standard error (n = 3).

Figure 5a shows the variation of *S*_11_*+S*_12 _with time for the three SGV values considered (6.9, 7.5, and 10.6 cm/min) at a pH of 5.5. As the gas flow rate increases the magnitude of *S*_11_*+S*_12 _is observed to decrease. If it is assumed that the column is operating in steady state for the period of experiment, the following relationships hold according to Stevenson [19] for a rising froth:(4)

Where, *j *is the SGV and the subscripts *f*, *g*, and *d *correspond to foamate, gas, and drainage. *R*_*b *_is the average bubble radius (in accordance to the equal volume sphere diameter), *r *is the radius of curvature of the plateau borders, and *Φ*_*l *_is the liquid fraction in foam. From the equations above it is clear that as the SGV (equivalent to gas flow rate for a constant diameter column) increases the liquid uptake in the foam. Further, this increase in liquid uptake inhibits coarsening (where gas diffuses from smaller to larger bubbles) and coalescence effects by decreasing the film surface area. This results in smaller bubbles with high liquid hold up. On the other hand, at lower gas velocities the liquid uptake is lower resulting in enhanced drainage with increased coarsening and coalescence. Thus, the foam is drier with larger bubble size, which should mean a higher enrichment. Indeed, this can be observed in Figure 5b. Also, larger bubble size and lower liquid content at lower flow rates leads to higher magnitudes of *S*_11_*+S*_12 _as expected [27].

It is important to emphasize here that the light scattering data exhibits significant noise. The foam flows as the measurements are taken; as a result, the foam layer in the detection plane at any given point in time is different from any other. The steady state, plug-flow nature of the process only ensures the similarity in foam layers statistically, while the scattered intensity shows a speckled pattern. Thus, a variation in *S*_11_*+S*_12 _is expected over time. Further, the utility of such a diagnostic scheme lies in being able to sense such variations.

### Correlation between *S*_11_+*S*_12 _and Enrichment

The light scattering data in Figure 4 and Figure 5 indicate that enrichment is higher when the magnitude of observed *S*_11_*+S*_12 _is higher. This points towards a direct proportionality between the enrichment and *S*_11_*+S*_12 _value. To investigate this further, out of the three replications performed (i.e. three sets of data obtained) for each experiment; two sets of data are used to perform a linear regression between *S*_11_*+S*_12 _and enrichment, and the third set is compared with predictions from the regression curve. Separate regression curves are obtained for the case of pH and flow rate variation, as they affect the foam properties in different ways. The *S*_11_*+S*_12 _data was averaged over the period (i.e. the time interval of 15 minutes) for which the enrichment ratio is computed. This mean value is compared with enrichment to perform the linear regression.

**Figure 6:**
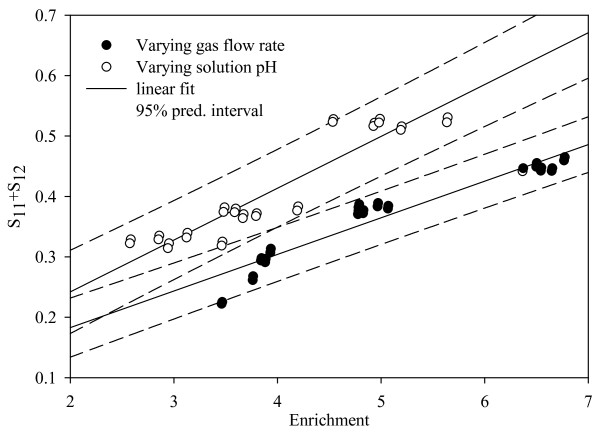
**Correlation between *S*_11_*+S*_12 _and enrichment for varying superficial gas velocity and varying pH values**.

Figure 6 shows the linear correlation between *S*_11_*+S*_12 _and enrichment, along with the 95% confidence and prediction intervals for the regression curve obtained. From the ANOVA the data corresponding to variation in SGV (R^2 ^= 0.9186, SE = 0.0216) provided a better goodness of fit when compared with the regression curve obtained for a variation in pH (R^2 ^= 0.8672, SE = 0.0314). Qualitatively, this is expected as flow rate has a rather direct influence on the foam properties in comparison to pH. The slope of the linear models for both the cases was significant (p < 0.0001) while the intercept has wider confidence limits and the standard error was comparable to its order of magnitude (p = 0.0016 for varying SGV and p = 0.0104 for varying pH).

**Figure 7:**
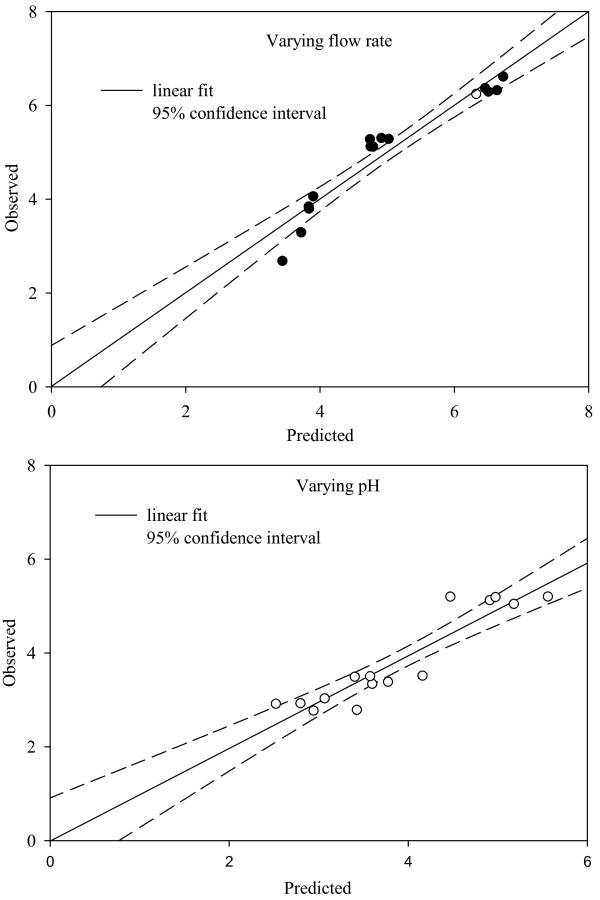
**Correlation between the observed and the predicted enrichment**. A linear model was used at varying superficial gas velocity and varying pH.

To investigate the predictive power of the linear fit obtained for the flow rate, an analysis was performed to determine if there was a significant difference between the predicted and observed enrichment from the third set of data (which was excluded while obtaining the linear fit). The observed enrichment was fit with respect to the enrichment values predicted from the *S*_11_*+S*_12 _data and the null hypothesis Ho: intercept = 0 and slope = 1 was tested by looking at the 95% confidence interval for the slope and the intercept. Figure 7a shows the comparison between the observed and predicted enrichment along with the regression line. The regression statistics show that the 95% confidence intervals for the slope and intercept contain the values 1 and 0, respectively, suggesting that the null hypothesis cannot be rejected. Hence, the observed and predicted values can be considered statistically not different. The standard error (SE) was found to be 0.348. A similar analysis was done using the linear fit obtained for data corresponding to variation in pH and the results are presented in Figure 7b. Although the linear fit is inferior in comparison to the flow rate data, the confidence intervals of the slope and the intercept contain the values 1 and 0, respectively, and the SE is 0.366.

### Response to Perturbation

**Figure 8:**
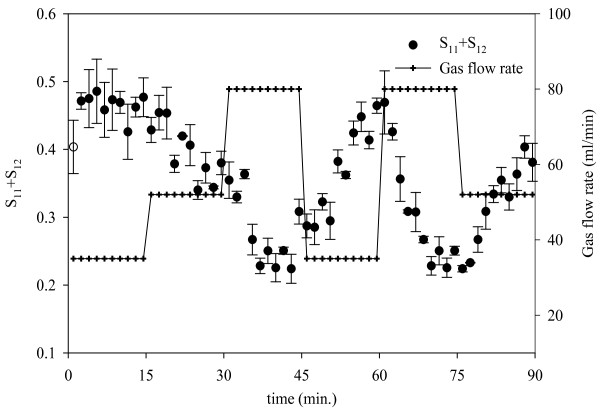
**Response of *S*_11_*+S*_12 _to step changes in superficial gas velocity**. Error bars are based on standard error (n = 3).

Figure 8 shows the response observed in the light scattering signal (S_11_+S_12_) over a period of 90 minutes during which the gas flow rate is varied every 15 minutes in steps. It is observed that there is a certain time lag before the variation is reflected in the intensity data. This is expected because the properties of the foam layer, where the light scattering measurements are taken, would change only after a time lag. This time lag is expected to be proportional to the foam overflow rate.

## Conclusions

The utility of polarized light scattering to monitor the performance of a foam fractionation column was investigated. The effect of pH and flow rate on the performance was measured and used to correlate the *S*_11_*+S*_12 _to enrichment ratio. The time average *S*_11_*+S*_12 _increases as the enrichment increases. This is attributed to the fact that larger bubbles with smaller liquid hold up lead to increased values of *S*_11_*+S*_12 _and higher enrichment [7]. The opposite is true when the bubbles are smaller with a higher liquid hold up. The linear model obtained from correlating the scattering data to the enrichment provides reasonable predictions but is not sufficient to estimate the enrichment ratio accurately. It is important to note that light scattering data and the enrichment are connected by the foam properties. Thus, knowledge of the nature of dependence between foam properties and *S*_11_*+S*_12 _combined with models relating the enrichment to the bubble size and liquid hold up is important to develop an accurate model relating *S*_11_*+S*_12 _with enrichment. On a general basis, it is understood that the parameter *S*_11_*+S*_12 _has a linear relationship with the bubble size and an inverse relationship with the liquid fraction. But the relation between enrichment and bubble size/liquid hold are complicated by a lack of understanding of protein/surfactant distribution within the foam.

## Competing interests

The authors declare that they have no competing interests.

## Authors' contributions

JNS designed the foaming column and conducted experiments. Data analysis was performed by JNS and CC. CC and MPM conceived of the study and participated in its design and coordination. All authors contributed to the writing of the manuscript and approved the final version.
